# ARID1a Gene as a Potential Early Marker to Tackle Endometriosis-Associated Ovarian Cancer

**DOI:** 10.14336/AD.2023.1109

**Published:** 2023-11-17

**Authors:** Pawel Kordowitzki, Sylvia Mechsner, Jalid Sehouli

**Affiliations:** ^1^Department of Preclinical and Basic Sciences, Nicolaus Copernicus University, Torun, Poland.; ^2^Department of Gynecology including Center of Oncological Surgery (CVK) and Department of Gynecology (CBF), European Competence Center for Ovarian Cancer, Charite, Berlin, Germany.

**Keywords:** endometriosis, epithelial ovarian cancer, endometrioid ovarian cancer, ARID1A mutation

## Abstract

The worries of women with endometriosis - a chronic gynecological disease affecting approximately 10% of women of childbearing age - about the increased risk of ovarian cancer are present worldwide. Endometriosis is a common, often painful, but benign gynecological disease that affects women. However, the pathogenesis remains elusive but is certainly multifactorial. Interestingly, endometriosis shares similarities with cancer. Therefore, women suffering from endometriosis fear an increased risk of ovarian cancer. In addition, these patients suffer from anxiety and depression. Previous studies have provided evidence that epithelial mutations in endometriosis or in the endometrium include certain inactivating mutations responsible for ovarian cancer, such as in the ARID1A gene.

The fear and concerns of women with endometriosis, a chronic gynecological condition affecting approximately 176 million (~10%) women of reproductive age worldwide, about the increased ovarian cancer risk are still up to date [1-3]. Endometriosis is a prevalent, often painful, but benign gynecological disorder that affects women of reproductive age. Its pathogenesis remains elusive but is surely multifactorial [3]. However, endometriosis shares features with cancer. Consequently, women suffering from endometriosis are concerned about the increased ovarian cancer risk, and endometriosis-associated ovarian cancer is challenging for clinicians [1-3]. Moreover, the patients are suffering from anxiety and depression, impacting their psychological and social functioning. These psychiatric issues should not be neglected since they again influence the level of the severity of endometriosis symptoms thus leading to a vicious cycle [4]. Furthermore, exposure to pain during endometriosis was estimated to be a primary factor for an elevated incidence of depression. As numerous patients report frequently, it is very hard to live with chronic pain since it affects performing all-day duties, hobbies, or tasks related to women’s jobs. Interestingly, the risk in women suffering from endometriosis to develop ovarian cancer is lower than 2%, compared to the 1-3% risk of ovarian cancer in the entire female population. Based on this, for women with endometriosis, the ovarian cancer risk is relatively low which should reduce women’s fear of ovarian cancer [3]. ARID1A is described as an epigenetic tumor suppressor, a component of the so-called SWI/SNF chromatin remodeling complexes, and very frequently- inactivating mutations of this gene are present in more than 50% of ovarian clear cell carcinomas, and approximately 30% of ovarian endometrioid carcinomas [5, 6]. Noteworthy, previous studies provided evidence that epithelial mutations in endometriosis or endometrium included distinct driver mutations for ovarian cancer such as in the ARID1A gene [1-3]. At first glance, these findings were puzzling because the direct observation of the malignant transformation of endometriotic lesions, particularly in the extra ovarian locations, has rarely been reported in the literature [1]. As mentioned above, ARID1A mutation is considered one of the most important driver events in endometriosis-associated ovarian cancer [2,3]. This raises the question regarding the significance of mutations in the ARID1A gene as an earlier biomarker in endometriosis patients to tackle ovarian cancer initiation, as partial loss of BAF250a expression has been found in lesions of patients with endometriosis [7]. In consequence, a patient with a history of endometriosis has a significantly increased risk of clear-cell and endometrioid ovarian cancer, which underlines the importance of deciphering the code for comorbidity. However, one should keep in mind that most women with endometriosis will rarely develop high-grade ovarian cancer, and that serous borderline tumours from which invasive low-grade serous ovarian cancers are believed to arise are not necessarily associated with a history of endometriosis [7]. Another possible explanation is that several molecular, local environmental, or epigenetic factors might give rise to invasive low-grade ovarian cancer, and endometriosis's effect is entirely independent of the association with serous borderline tumours. Results of molecular studies have highlighted the presence of ARID1A gene mutations in 46% of clear-cell and 30% of endometrioid ovarian cancers and in areas of endometriosis that are contiguous with these cancers [8,9]. In this regard, it appears worth suggesting ovarian cancer screening for those patients with an endometriosis history to shed light on the pathogenesis of endometriosis-associated ovarian cancer. Importantly though, biomarker research, including mutation analyses of the ARID1A gene, early detection of activated oncogenes, and the silencing of tumor suppressors are strongly advised. In conclusion, we suggest that doctors should reassure women suffering from endometriosis that the risk of developing ovarian cancer is low and that early care for the patient’s mental well-being, for instance, psychological support groups, or resources for managing anxiety and fear, should be recommended by clinicians. Counseling programs for the patients should also be provided to test for ARID1a mutations. Another important practical consideration in regard to the clinical implications of endometriosis-associated ovarian cancer should focus on fertility preservation, especially nowadays since couples tend to delay the age of the first pregnancy. The earlier-mentioned counseling programs for affected women should also contain the advantages and disadvantages of assisted reproductive technologies, such as the cryopreservation of oocytes as a potential clinical intervention to preserve fertility [10].

**Figure 1.**
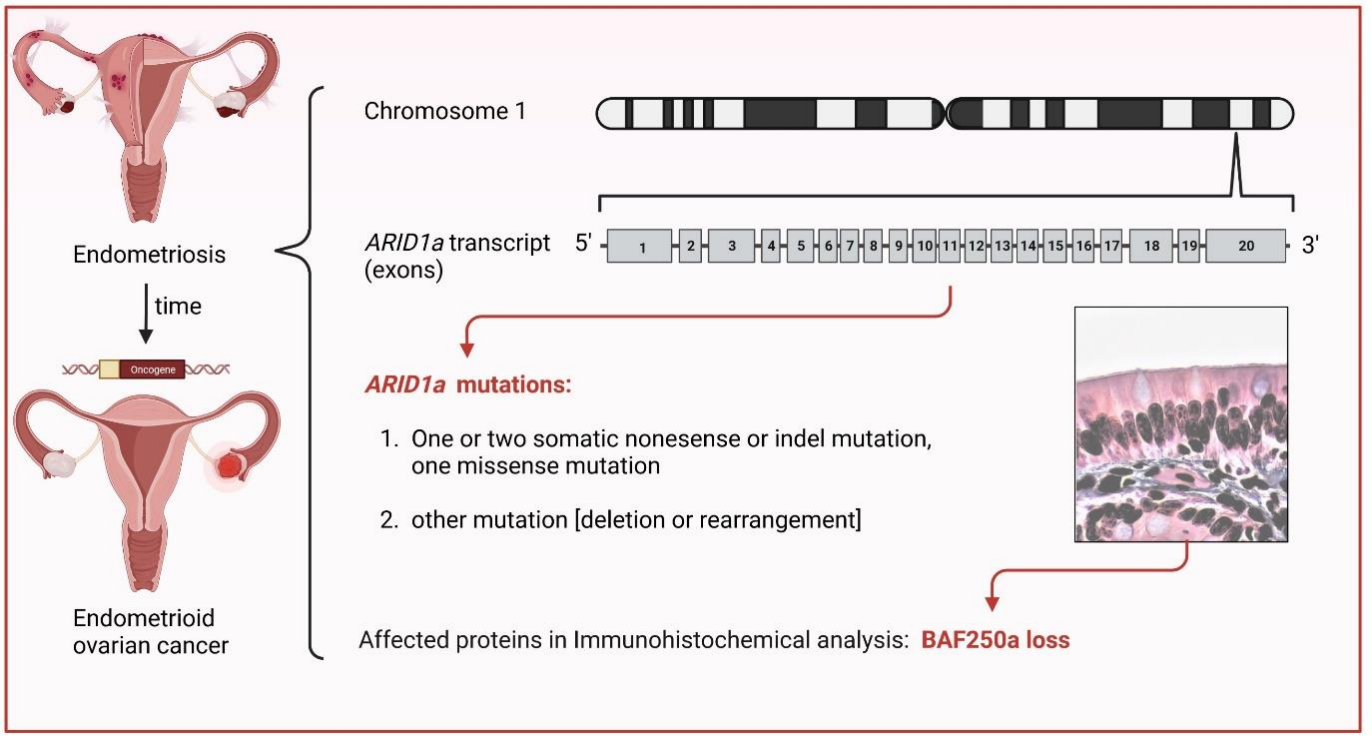
Schematic representation of *ARID1a* mutation as potential early marker for malignant transformation of endometriosis and endometroid ovarian cancer initiation in women.

## References

[1] Anglesio MS, Papadopoulos N, Ayhan A, Nazeran TM, Noë M, Horlings HM et al. (2017). Cancer-Associated Mutations in Endometriosis without Cancer. N Engl [J] Med. 376(19):1835-1848. 10.1056/NEJMoa1614814 PMC5555376 28489996

[2] Fonseca MAS, Haro M, Wright KN, Lin X, Abbasi F, Sun J et al. (2023). Single-cell transcriptomic analysis of endometriosis. Nat Genet 55, 255-267 (2023). 36624343 10.1038/s41588-022-01254-1 PMC10950360

[3] Kvaskoff M, Horne AW, Missmer SA (2017). Informing women with endometriosis about ovarian cancer risk. Lancet. 390(10111):2433-2434. 29208299 10.1016/S0140-6736(17)33049-0

[4] Laganà AS, La Rosa VL, Rapisarda AMC, Valenti G, Sapia F, Chiofalo B, et al. (2017). Anxiety and depression in patients with endometriosis: impact and management challenges. Int [J] Womens Health. 9:323-330. 10.2147/IJWH.S119729 PMC5440042 28553145

[5] Kinose Y, Xu H, Kim H, Kumar S, Shan X, George E, et al.(2023). Dual blockade of BRD4 and ATR/WEE1 pathways exploits ARID1A loss in clear cell ovarian cancer. Res Sq, 27:rs.3.rs-3314138.

[6] Borrelli GM, Abrão MS, Taube ET, Darb-Esfahani S, Köhler C, Kaufmann AM, et al. (2015). Immunohistochemical Investigation of Metastasis-Related Chemokines in Deep-Infiltrating Endometriosis and Compromised Pelvic Sentinel Lymph Nodes. Reprod Sci. 22(12):1632-42. 26169037 10.1177/1933719115592711

[7] Wu JN, Roberts CW (2023). ARID1A mutations in cancer: another epigenetic tumor suppressor? Cancer Discov. 3(1):35-43. 10.1158/2159-8290.CD-12-0361 PMC3546152 23208470

[8] Gourley C (2012). Link between endometriosis and ovarian-cancer subtypes. Lancet Oncol. 13(4):326-8. 22361335 10.1016/S1470-2045(12)70029-3

[9] Wiegand KC, Shah SP, Al-Agha OM, Zhao Y, Tse K, Zeng T et al. (2010). ARID1A mutations in endometriosis-associated ovarian carcinomas. N Engl J Med; 363: 1532-43. 20942669 10.1056/NEJMoa1008433 PMC2976679

[10] Younis JS (2022). Endometriosis-Associated Ovarian Cancer: What Are the Implications for Women with Intact Endometrioma Planning for a Future Pregnancy? A Reproductive Clinical Outlook. Biomolecules. 21;12(11):1721. 36421735 10.3390/biom12111721 PMC9688199

